# Prognostic scoring system based on eosinophil- and basophil-related markers for predicting the prognosis of patients with stage II and stage III colorectal cancer: a retrospective cohort study

**DOI:** 10.3389/fonc.2023.1182944

**Published:** 2023-07-14

**Authors:** Lijing Gao, Chao Yuan, Jinming Fu, Tian Tian, Hao Huang, Lei Zhang, Dapeng Li, Yupeng Liu, Shuhan Meng, Ying Liu, Yuanyuan Zhang, Jing Xu, Chenyang Jia, Ding Zhang, Ting Zheng, Qingzhen Fu, Shiheng Tan, Li Lan, Chao Yang, Yashuang Zhao, Yanlong Liu

**Affiliations:** ^1^ Department of Epidemiology, School of Public Health, NHC Key Laboratory of Etiology and Epidemiology (23618504), Harbin Medical University, Harbin, Heilongjiang, China; ^2^ Department of Colorectal Surgery, Harbin Medical University Cancer Hospital, Harbin, Heilongjiang, China; ^3^ Division of Chronic and Non-communicable Diseases, Harbin Center for Diseases Control and Prevention, Harbin, Heilongjiang, China

**Keywords:** colorectal cancer, prognosis, biomarker, inflammation, eosinophils, basophils, risk score

## Abstract

**Background:**

Systemic inflammation is associated with the prognosis of colorectal cancer (CRC). The current study aimed to construct a comprehensively inflammatory prognostic scoring system named risk score (RS) based on eosinophil- and basophil-related markers and assess its prognostic value in patients with stage II and stage III CRC.

**Patients and methods:**

A total of 3,986 patients were enrolled from January 2007 to December 2013. The last follow-up time was January 2019. They were randomly assigned to the training set and testing set in a 3:2 split ratio. Least absolute shrinkage and selection operator (LASSO)–Cox regression analysis was performed to select the optimal prognostic factors in the construction of RS. The Kaplan–Meier curve, time-dependent receiver operating characteristic (ROC), and Cox analysis were used to evaluate the association between RS and overall survival (OS).

**Results:**

In the training set, all inflammatory markers showed certain prognostic values. Based on LASSO-Cox analysis, nine markers were integrated to construct RS. The Kaplan–Meier curve showed that a higher RS (RS > 0) had a significantly worse prognosis (log-rank *p*< 0.0001). RS (>0) remained an independent prognostic factor for OS (hazard ratio (HR): 1.70, 95% confidence interval (CI), 1.43–2.03, *p*< 0.001). The prognostic value of RS was validated in the entire cohort. Time-dependent ROC analysis showed that RS had a stable prognostic effect throughout the follow-up times and could enhance the prognostic ability of the stage by combination. Nomogram was established based on RS and clinicopathological factors for predicting OS in the training set and validated in the testing set. The area under the curve (AUC) values of the 3-year OS in the training and testing sets were 0.748 and 0.720, respectively. The nomogram had a satisfactory predictive accuracy and had better clinical application value than the tumor stage alone.

**Conclusions:**

RS might be an independent prognostic factor for OS in patients with stage II and III CRC, which is helpful for risk stratification of patients. Additionally, the nomogram might be used for personalized prediction and might contribute to formulating a better clinical treatment plan.

## 1 Introduction

Colorectal cancer (CRC), including colon cancer and rectal cancer, is one of the most common malignant tumors threatening human health. Based on the GLOBOCAN 2020 estimation (1), CRC is the second leading cause of death, only next to lung cancer, with more than 935,000 deaths. There were 51,020 CRC deaths in the United States in 2019, equivalent to 8.4% of all cancer deaths (2). In China, 191,000 patients died of CRC, which ranks fifth in cancer deaths in 2015 (3). Surgical resection is the most common therapy for patients with CRC, and tumor stage based on pathological characteristics is widely used for evaluating the prognosis of CRC patients (4, 5). However, studies have shown that the prognosis with the same stage varies greatly and is highly heterogeneous (6). In addition, the pathological characteristics are mainly obtained by biopsy or pathological reports, which are hard to represent the overall condition of the tumor. Thus, it is necessary to identify a non-invasive and more accurate marker to assess the prognosis of CRC patients.

Recently, increasing evidence has demonstrated that systemic inflammation is closely associated with the progression and prognosis of CRC (7–9). The inflammatory markers based on peripheral leukocytes include neutrophil, lymphocyte, and monocyte counts, and related indicators such as neutrophil-to-lymphocyte ratio and lymphocyte-to-monocyte ratio, which have been widely studied to predict the prognosis of patients with CRC as well as other types of malignant tumors (10–15). Eosinophils and basophils account for a small proportion of circulating leukocytes in the bloodstream. To date, little is known about the prognostic impact of eosinophil- and basophil-related markers in CRC patients. In 2018, Wei et al. first reported that circulating hypoeosinophilia and basophilia were associated with worse prognosis in patients with CRC in 569 samples (16). In addition, studies have shown that tumor eosinophilia and basophilia infiltrations contribute to predicting the survival of cancer patients (17–19). To our knowledge, the eosinophil-to-neutrophil ratio (20, 21), eosinophil-to-basophil ratio (22), monocyte-to-eosinophil ratio (23, 24), eosinophil-to-lymphocyte ratio (25), and basophil-to-lymphocyte ratio (26) have mainly been reported in inflammatory diseases. However, the relationship between these inflammatory markers and the prognosis of patients with stage II and stage III CRC is indistinct. Additionally, compared to a single type marker, a combination of them might be more valuable and could provide more accurate information for prognosis.

Thus, our study comprehensively analyzed and integrated these inflammatory markers based on eosinophil- and basophil-related markers by least absolute shrinkage and selection operator (LASSO)–Cox regression analysis and constructed a prognostic scoring system named risk score (RS) in CRC. We evaluated the prognostic value of RS for stage II and stage III CRC patients. Additionally, the development and validation of a nomogram for personalized survival prediction might contribute to formulating a better clinical treatment plan.

## 2 Patients and methods

### 2.1 Patients and study design

**Figure 1:**
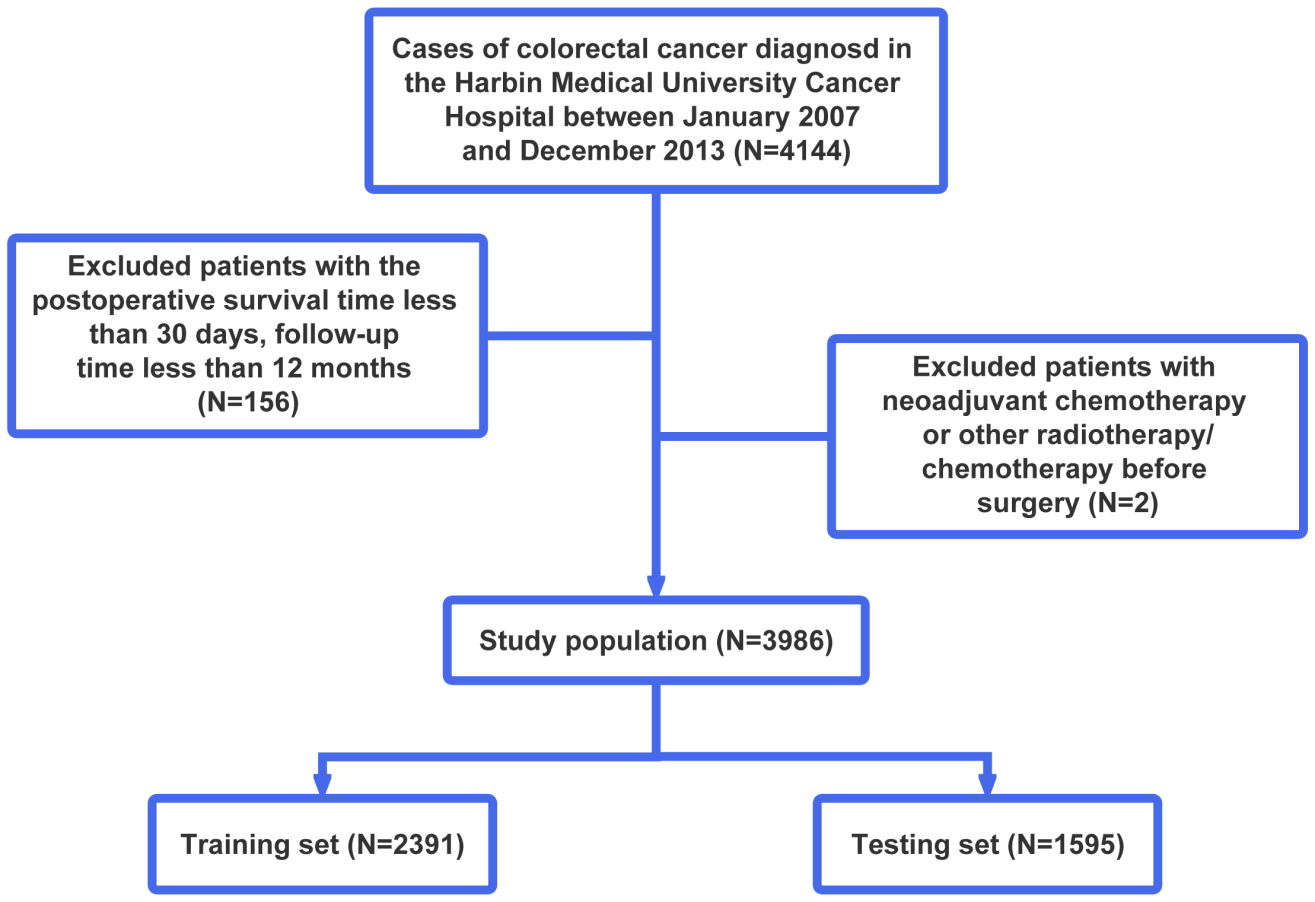
Flowchart of patient selection. According to the exclusion criteria, a total of 3,986 patients were included in this study, and they were randomly allocated into the training and testing sets in a 3:2 ratio.

A total of 4,144 primary stage II and III CRC patients undergoing surgical resection followed by pathological diagnosis at the Harbin Medical University Cancer Hospital between January 2007 and December 2013 were enrolled in this study. We excluded patients with neoadjuvant chemotherapy or other radiotherapy/chemotherapy before surgery (n = 2), a postoperative survival time of less than 30 days, or a follow-up time of less than 12 months (n = 156). Finally, 3,986 CRC patients were included in further analyses. Then, they were randomly assigned to the training set and testing set in a 3:2 split ratio. The training set consisting of 2,391 patients was used to train our model, while the testing set consisting of the remaining 1,595 patients was used to evaluate the performance and generalizability of the model after it had been trained. The flowchart of patient screening is shown in **Figure 1**. This study complied with the standards of the Declaration of Helsinki.

### 2.2 Data collection

Patients’ clinicopathological features and routine blood results were retrieved from the medical records. The clinicopathological features include sex, age, tumor location, gross appearance, differentiation degree, histological type, tumor stage, cancer nodes, perineural invasion, neoplastic thrombosis, postoperative chemotherapy, and postoperative radiotherapy. Blood routine tests were based on a single blood sample of each patient, which was measured by an autoanalyzer (Sysmex XE-2100, Kobe, Japan). Follow-up information was obtained retrospectively through electronic medical records and telephone interviews. The last time of follow-up was in January 2019. The overall survival (OS) was defined as the time from surgery to death from any cause or the last follow-up visit.

### 2.3 Inflammation biomarkers

Data on peripheral white blood cell (W; 10^9^/L), neutrophil (N; 10^9^/L), lymphocyte (L; 10^9^/L), monocyte (M; 10^9^/L), eosinophil (E; 10^9^/L), basophil (B; 10^9^/L), and platelet (P; 10^9^/L) were extracted from the results of the first blood routine tests (limit to 30 days before surgery). When the absolute numbers of basophils and eosinophils were 0 (10^9^/L), we added 0.0001 (10^9^/L) to enable them to calculate ratios. Inflammatory factors based on eosinophils and basophils, including EBR, NER, MER, LER, WER, PER, NBR, MBR, LBR, WBR, and PBR were calculated as follows: EBR = E/B, NER = N/E, MER = M/E, LER = L/E, WER = W/E, PER = P/E, NBR = N/B, MBR = M/B, LBR = L/B, WBR = W/B, and PBR = P/B.

### 2.4 Development of an inflammatory prognostic scoring system

The optimal cutoff values of the above 11 inflammatory markers for predicting the overall survival of patients with CRC were identified by X-tile 3.6.1 software (27) (Yale University, New Haven, CT, USA), which were then classified as categorical variables according to the cutoff value. The variables below and above the cutoff values were scored 0 and 1, respectively. Pearson’s correlation method was used to calculate correlation coefficients for inflammatory biomarkers. Considering the existence of multicollinearity among them, the LASSO-Cox regression analysis with 10-fold cross-validation was performed to select the optimal prognostic factors using the R package “glmnet” and “survminer”. The inflammatory biomarkers with non-zero coefficients were incorporated to construct the novel RS, which was calculated as follows:


$$\text{Risk score (RS)}=\sum_{i=1}^{n}\left(Score\times Coef\right)$$

Here, *n* represents the number of inflammatory markers, *Score* is the score of each inflammatory marker, *Coef* is the coefficient of LASSO-Cox regression analysis, and risk score (RS) represents a weighted sum of the prognosis score of each marker. RS was also divided into two groups (low and high) by X-tile software in the training set. A time-dependent receiver operating characteristic (ROC) curve was performed to evaluate the predictive value of RS for OS in CRC patients by the R package “timeROC”.

### 2.5 Statistical analysis

Continuous data were shown as median (interquartile range [IQR]), whereas categorical variables were reported as numbers and percentages. The Wilcoxon rank sum test and Pearson’s chi-squared test were used to compare the clinicopathological characteristics of the training and testing cohorts. The Kaplan–Meier and log-rank tests were utilized to generate the survival curves and compare the survival differences among the groups. The univariate and multivariate Cox analyses were used to estimate the association between indicators and OS, and the results were presented as hazard ratio (HR) and 95% confidence interval (CI). The subgroup analysis was conducted, stratified by sex, age, tumor location, gross appearance, differentiation degree, histological type, tumor stage, perineural invasion, postoperative chemotherapy, and postoperative radiotherapy in the training set.

A nomogram was established by the independent prognostic factors according to multivariate Cox analysis in the training set through the package “rms” in R software. The performance of the nomogram was evaluated by the concordance index (C-index) and time-dependent ROC curve. The C-index >0.5 indicates that the model could discriminate the outcome. The closer the value of the C-index approached 1.0, the higher the prognostic accuracy. Finally, 1,000 bootstrap resamples were performed for internal validation. The performance of nomograms was explored also through calibration curves and decision curve analysis (DCA).

Statistical analyses were conducted with SPSS 23.0 software (SPSS, Inc., Chicago, IL, USA) and R Studio version 3.6.3. All statistical tests were two-sided, and a *p*-value<0.05 was considered statistically significant.

## 3 Results

### 3.1 Baseline characteristics of patients

A total of 3,986 patients with stage II and III CRC were included in this study; the population was randomly divided into the training set (2,391 patients) and the testing set (1,595 patients). The longest follow-up time was nearly 144 months. Detailed baseline characteristics of each set are described in **Table 1**. In the training set, the median follow-up time was 73 months (IQR, 56–95.5 months), and the median OS time was 68 months (IQR, 44–92 months). In the testing set, the median follow-up time was 71 months (IQR, 56–94 months), and the median OS time was 67 months (IQR, 45–92 months). There were no significant differences between the training set and the testing set (*p* > 0.05), indicating that the division of data was balanced (**Table 1**).

**Table 1:** Baseline characteristics of CRC patients in the training set and testing set.

Characteristic	Overall, n = 3,986	Training set, n = 2,391	Testing set, n = 1,595	*p*-Value^1^
Time, median (IQR)	68.00 (45.00, 92.00)	68.00 (44.00, 92.00)	67.00 (45.00, 92.00)	>0.90
Sex, n (%)				0.09
Female	2,361 (59%)	1,442 (60%)	919 (58%)	
Male	1,625 (41%)	949 (40%)	676 (42%)	
Age, n (%)				**0.03**
<60	2,173 (55%)	1,270 (53%)	903 (57%)	
≥60	1,813 (45%)	1,121 (47%)	692 (43%)	
Location, n (%)				>0.90
Colon cancer	1,895 (48%)	1,136 (48%)	759 (48%)	
Rectal cancer	2,091 (52%)	1,255 (52%)	836 (52%)	
Gross appearance, n (%)				0.20
Bulge	2,654 (67%)	1,575 (66%)	1,079 (68%)	
Infiltration or ulcer	1,332 (33%)	816 (34%)	516 (32%)	
Differentiation degree, n (%)				0.13
Poor	532 (13%)	335 (14%)	197 (12%)	
Moderate or well	3,454 (87%)	2,056 (86%)	1,398 (88%)	
Histological type, n (%)				0.30
Adenocarcinoma	3,022 (76%)	1,798 (75%)	1,224 (77%)	
Mucinous adenocarcinoma or signet ring cell cancer	964 (24%)	593 (25%)	371 (23%)	
Tumor stage, n (%)				0.40
Stage II	2,348 (59%)	1,396 (58%)	952 (60%)	
Stage III	1,638 (41%)	995 (42%)	643 (40%)	
Cancer nodes, n (%)				0.90
No	3,723 (93%)	2,232 (93%)	1,491 (93%)	
Yes	263 (6.6%)	159 (6.6%)	104 (6.5%)	
Perineural invasion, n (%)				0.08
No	3,688 (93%)	2,198 (92%)	1,490 (93%)	
Yes	298 (7.5%)	193 (8.1%)	105 (6.6%)	
Neoplastic thrombosis, n (%)				0.40
No	3,857 (97%)	2,309 (97%)	1,548 (97%)	
Yes	129 (3.2%)	82 (3.4%)	47 (2.9%)	
Postoperative chemotherapy, n (%)				0.70
No	2,279 (57%)	1,372 (57%)	907 (57%)	
Yes	1,707 (43%)	1,019 (43%)	688 (43%)	
Postoperative radiotherapy, n (%)				0.80
No	3,807 (96%)	2,282 (95%)	1,525 (96%)	
Yes	179 (4.5%)	109 (4.6%)	70 (4.4%)	
WER, median (IQR)	53.71 (31.83, 96.97)	52.60 (31.98, 96.93)	54.31 (31.53, 98.85)	>0.90
PER, median (IQR)	2,129.46 (1,189.93, 4,087.11)	2,101.56 (1,192.09, 4,023.50)	2,160.08 (1,180.57, 4,242.35)	0.70
LER, median (IQR)	16.15 (9.53, 29.17)	16.18 (9.56, 28.93)	16.10 (9.52, 29.77)	0.70
NER, median (IQR)	30.99 (17.91, 59.47)	30.65 (18.06, 59.78)	31.60 (17.85, 58.69)	>0.90
MER, median (IQR)	3.57 (2.09, 6.58)	3.55 (2.07, 6.58)	3.59 (2.13, 6.59)	0.50
EBR, median (IQR)	3.30 (1.83, 6.60)	3.36 (1.89, 6.61)	3.21 (1.76, 6.51)	0.40
WBR, median (IQR)	164.11 (108.51, 301.65)	166.17 (108.58, 300.83)	161.35 (108.12, 302.22)	0.80
PBR, median (IQR)	6,361.04 (4,192.57, 12,006.08)	6,425.99 (4,235.56, 11,969.20)	6,283.52 (4,174.27, 12,160.95)	0.80
LBR, median (IQR)	46.96 (31.85, 84.18)	46.78 (31.77, 83.96)	47.27 (31.98, 84.56)	0.80
NBR, median (IQR)	99.00 (61.83, 192.43)	100.47 (62.30, 187.65)	96.50 (61.41, 200.11)	0.60
MBR, median (IQR)	11.22 (7.44, 20.70)	11.37 (7.37, 20.19)	11.13 (7.56, 21.25)	0.60

Wilcoxon rank sum test; Pearson’s chi-squared test.

CRC, colorectal cancer; IQR, interquartile range; WER, white blood cell-to-eosinophil ratio; PER, platelet-to-eosinophil ratio; LER, lymphocyte-to-eosinophil ratio; NER, neutrophil-to-eosinophil ratio; MER, monocyte-to-eosinophil ratio; EBR, eosinophil-to-basophil ratio; WBR, white blood cell-to-basophil ratio; PBR, platelet-to-basophil ratio; LBR, lymphocyte-to-basophil ratio; NBR, neutrophil-to-basophil ratio; MBR, monocyte-to-basophil ratio.

^1^Bold indicates significance (*p*-value<0.05).

### 3.2 Optimal cutoff values of inflammatory biomarkers for predicting the overall survival of CRC

We determined the optimal cutoff values of inflammatory biomarkers in the training set by X-tile software (**Supplementary Figures 1–3**), in which patients were divided into low and high groups. Univariate Cox analyses for OS of inflammatory factors in the training set showed that higher pretreatment EBR (*p* = 0.01), PBR (*p* = 0.003), and LBR (*p*< 0.001) had significantly favorable OS probability than patients in the low groups, whereas others were accompanied by inferior OS (**Supplementary Table 1**).

### 3.3 Risk score construction for overall survival

**Figure 2:**
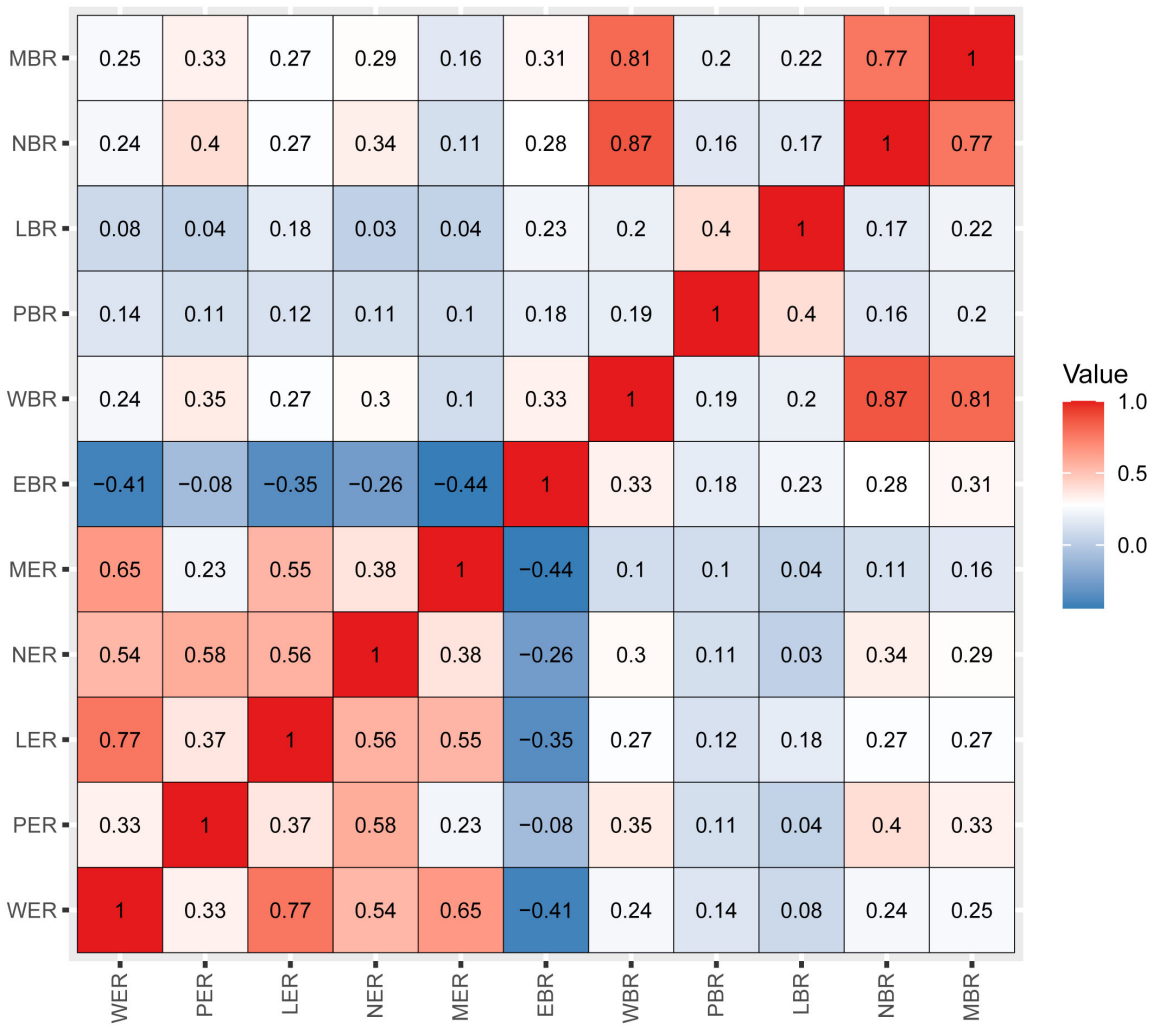
Pearson’s correlation coefficients among the 11 inflammatory markers. Blue indicates negative correlation, and red indicates positive correlation. Darker colors are associated with stronger correlation coefficients. WER, white blood cell-to-eosinophil ratio; PER, platelet-to-eosinophil ratio; LER, lymphocyte-to-eosinophil ratio; NER, neutrophil-to-eosinophil ratio; MER, monocyte-to-eosinophil ratio; EBR, eosinophil-to-basophil ratio; WBR, white blood cell-to-basophil ratio; PBR, platelet-to-basophil ratio; LBR, lymphocyte-to-basophil ratio; NBR, neutrophil-to-basophil ratio; MBR, monocyte-to-basophil ratio.

**Figure 3:**
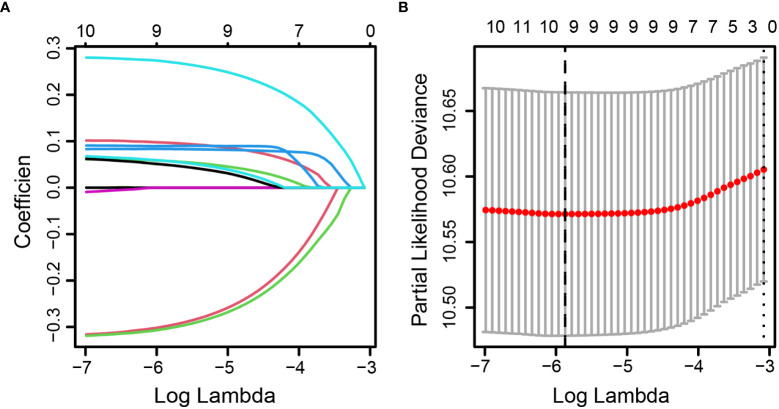
Identification of optimal inflammatory markers in colorectal cancer patients. Selection of optimal inflammatory markers in the LASSO model **(A)**. Tenfold cross-validation for tuning parameter (λ) selection in the LASSO model **(B)**. The dotted vertical lines were drawn at the optimal values using the maximum criteria and the one standard error of the maximum criteria. LASSO, least absolute shrinkage and selection operator.

**Figure 4:**
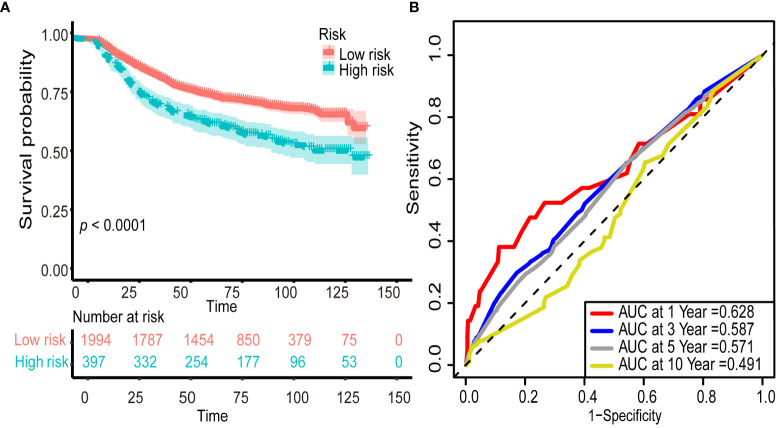
Predictive overall survival performance of risk score using Kaplan–Meier survival curve and time-dependent ROC analysis. The Kaplan–Meier survival curve showed that the overall survival probability in the low-risk group was significantly higher than that in the high-risk group (log-rank *p*< 0.0001; **(A)**. The prognostic accuracy of risk score was evaluated by the time-dependent ROC, yielding AUC values with 1-, 3-, 5-, and 10-year overall survival rates in the training set **(B)**. ROC, receiver operating characteristic; AUC, area under the curve.

Pearson’s correlation method was used to calculate correlation coefficients for the above 11 inflammatory markers, which showed a high correlation among the inflammatory markers (**Figure 2**). According to the results of univariate Cox analyses in the training set, inflammatory factors with *p*< 0.05 were included in the LASSO-Cox regression model; among the 11 candidate inflammatory biomarkers, PER, LER, NER, MER, WBR, PBR, LBR, NBR, and MBR were non-zero coefficients, and the optimal λ value = 0.0028, log (λ) = −5.8721 (**Figure 3**). The risk score was calculated based on the corresponding coefficient from LASSO, calculated as follows: RS = 0.0969 × PER + 0.0577 × LER + 0.0833 × NER + 0.2716 × MER + 0.0494 × WBR + (−0.2983) × PBR + (−0.3039) × LBR +0.0896 × NBR + 0.0577 × MBR. X-tile 3.6.1 software was also used to determine the optimal cutoff values for RS, which was 0 (**Supplementary Figure 4**). Patients were separated into the low-risk group (RS ≤ 0) and high-risk group (RS > 0) for further study. The Kaplan–Meier survival curve showed that the OS probability in the low-risk group was significantly higher than that in the high-risk group (log-rank *p*< 0.0001, **Figure 4A**). The prognostic accuracy of RS was evaluated by area under the curve (AUC) in the time-dependent ROC, yielding AUC values with 1-, 3-, 5-, and 10-year OS rates of 0.628, 0.587, 0.571, and 0.491, respectively, in the training set (**Figure 4B**).

### 3.4 Independent prognostic factors for CRC patients

In the training set, univariate Cox analysis showed that sex, age, tumor location, gross appearance, degree of differentiation, histological type, tumor stage, cancer nodes, perineural invasion, neoplastic thrombosis, postoperative chemotherapy, postoperative radiotherapy, eosinophils, basophils, and RS were associated with the prognosis of OS (all *p*< 0.1). All these statistically significant factors were then subjected to the multivariate Cox analysis. After adjustment of clinicopathological characteristics, eosinophils (HR: 0.75, 95% CI, 0.62–0.91) and basophils (HR: 0.81, 95% CI, 0.67–0.98) were still significantly associated with the OS of CRC (**Supplementary Table 2**). RS (high-risk *vs.* low-risk, HR: 1.70, 95% CI, 1.43–2.03) remained as an independent prognostic factor for poor OS (**Table 2**), which was further verified in the entire set (n = 3,986, HR: 1.44, 95% CI, 1.24–1.66). However, the result in the testing set was not significant (n = 1,595, **Supplementary Table 3**).

**Table 2:** Univariate and multivariate Cox analyses of baseline characteristics and risk score on overall survival in stage II and stage III colorectal cancer patients.

Characteristic	Univariate analyses			Multivariate analyses		
	HR	95% CI	*p*-Value	HR	95% CI	*p*-Value
Sex						
Male	1.00			1.00		
Female	0.87	0.75–1.02	0.08	0.83	0.71–0.97	0.021
Age						
<60	1.00			1.00		
≥60	1.64	1.41–1.91	<0.001	1.67	1.43–1.96	<0.001
Location						
Colon cancer	1.00			1.00		
Rectal cancer	1.31	1.13–1.52	<0.001	1.23	1.05–1.44	0.010
Gross appearance						
Bulge	1.00			1.00		
Infiltration or ulcer	1.47	1.26–1.71	<0.001	1.34	1.14–1.56	<0.001
Differentiation degree						
Poor	1.00			1.00		
Moderate or well	0.60	0.50–0.73	<0.001	0.70	0.58–0.85	<0.001
Histological type						
Adenocarcinoma	1.00			1.00		
Mucinous adenocarcinoma or signet ring cell cancer	1.23	1.04–1.46	0.015	1.35	1.14–1.60	<0.001
Tumor stage						
Stage II	1.00			1.00		
Stage III	2.27	1.95–2.64	<0.001	2.20	1.86–2.60	<0.001
Cancer nodes						
No	1.00			1.00		
Yes	2.09	1.61–2.70	<0.001	1.22	0.93–1.61	0.150
Perineural invasion						
No	1.00			1.00		
Yes	1.76	1.38–2.25	<0.001	1.44	1.11–1.86	0.006
Neoplastic thrombosis						
No	1.00			1.00		
Yes	2.15	1.53–3.00	<0.001	1.42	1.00–2.01	0.052
Postoperative chemotherapy						
No	1.00			1.00		
Yes	0.76	0.65–0.89	<0.001	0.66	0.56–0.79	<0.001
Postoperative radiotherapy						
No	1.00			1.00		
Yes	1.96	1.47–2.60	<0.001	1.79	1.33–2.41	<0.001
Risk score						
Low	1.00			1.00		
High	1.68	1.41–2.00	<0.001	1.70	1.43–2.03	<0.001

All analyses were adjusted for sex, age, tumor location, gross appearance, differentiation degree, histologic type, tumor stage, cancer nodes, perineural invasion, neoplastic thrombosis, postoperative chemotherapy, and postoperative radiotherapy.

### 3.5 Subgroup analysis

We investigated the prognostic effect of eosinophils, basophils, and RS in different subgroups stratified by sex, age, tumor location, gross appearance, differentiation degree, histological type, tumor stage, perineural invasion, postoperative chemotherapy, and postoperative radiotherapy in the training set. The results of subgroup analysis showed that both eosinophils and basophils were significantly associated with OS in the subgroups of<60 years, bulge, adenocarcinoma, no perineural invasion, no postoperative chemotherapy, and no postoperative radiotherapy (**Supplementary Figures 5, 6**). RS was still an independent prognostic factor for OS in all subgroups, except the poorly differentiated group, perineural invasion group, and postoperative radiotherapy group in the training set (**Supplementary Figure 7**).

### 3.6 The prognostic accuracy of risk score, TNM, their combination, and previously reported markers

We evaluated the prognostic accuracy of RS, TNM staging, their combination, and previously reported markers (neutrophil-to-lymphocyte ratio (NLR), lymphocyte-to-monocyte ratio (LMR), and platelet-to-lymphocyte ratio (PLR)) by AUC in the time-dependent ROC in the training set; the details of the AUC values are listed in **Supplementary Table 4**. RS had a stable prognostic effect, which tended to be higher than the NLR, LMR, and PLR throughout the follow-up times and could enhance the prognostic effect of the stage by the combination (**Supplementary Figure 8**).

### 3.7 Development and validation of the nomogram model for predicting the overall survival

**Figure 5:**
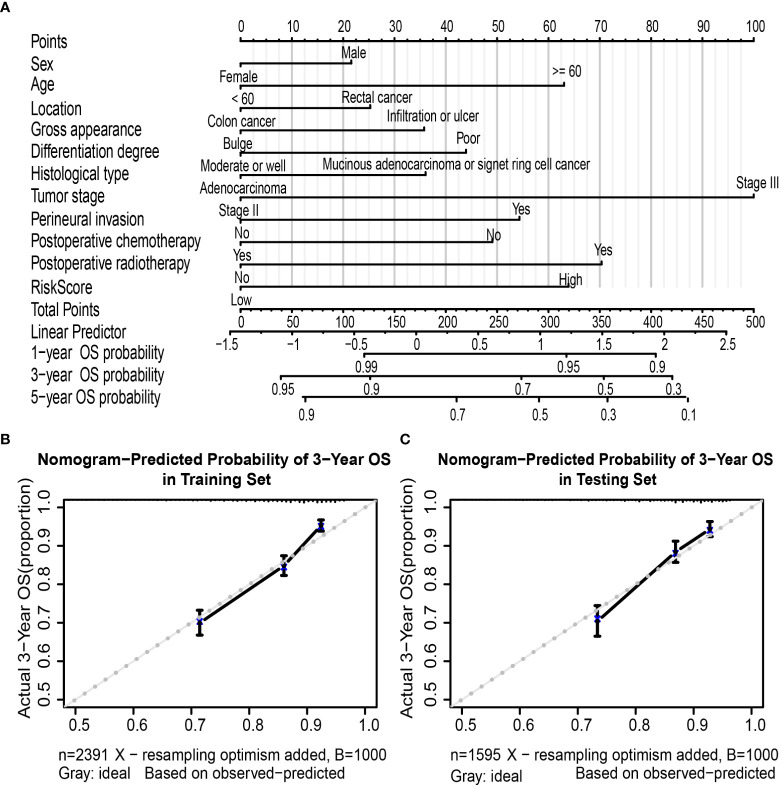
Nomogram to predict OS in colorectal cancer patients. Nomogram was performed by using risk score and clinical characteristics for predicting OS **(A)**. Calibration curves of the nomogram to predict OS at 3 years in the training set **(B)** and the testing set **(C)**. Nomogram can be interpreted by assigning points to each clinicopathological characteristic and risk score of patients at the top line and then summing up the points to predict the 1-, 3-, and 5-year OS probability of patients with CRC. Calibration curve; the y-axis represents the actual OS proportion, and the x-axis represents the nomogram-predicted probability of OS. The reference line is 45° and represents a perfect calibration by an ideal model. OS, overall survival; CRC, colorectal cancer.

A nomogram based on multivariate regression of the training set was further built; the nomogram was established by the above clinicopathological characteristics and RS (**Figure 5A**) by assigning points to each variable at the top line and then calculating the total points to predict 3-year OS probability or 5-year OS probability. For the training set, the nomogram’s C-index was 0.692 (95% CI, 0.672–0.712), which was the same as the bootstrapping method used in internal validation (0.692). For the testing set, the C-index was 0.691 (95% CI, 0.667–0.716) and also 0.691 in the internal validation. Moreover, the calibration curves of the nomogram indicated good agreement between the nomogram-predicted probability of 3 years and the actual 3-year OS proportion in the training set and testing set (**Figures 5B, C**).

Furthermore, the AUC values of the nomogram were higher than tumor stage in both training (3-year AUC: 0.748 *vs.* 0.680, **Supplementary Figure 9A**) and testing (3-year AUC: 0.720 *vs.* 0.648, **Supplementary Figure 9B**) cohorts for 3-year OS. Finally, DCA for 3-year OS prediction in the training (**Supplementary Figure 9C**) and testing (**Supplementary Figure 9D**) sets also showed favorable effects and had better clinical application value than the tumor stage alone.

## 4 Discussion

In this large retrospective cohort study, we constructed a novel RS by integrating the inflammatory markers selected by the LASSO analysis (PER, LER, NER, MER, WBR, PBR, LBR, NBR, and MBR). The multivariate Cox regression analysis revealed that RS was an independent prognostic factor for OS, and the high-risk group showed a significantly worse outcome in stage II and stage III CRC patients. RS had a stable prognostic ability at different follow-up times and could enhance the prognostic effect of tumor stage by combination. Furthermore, the nomogram constructed by RS and clinicopathological characteristics might be used for personalized prediction and help clinicians identify high-risk patients.

For CRC patients, the tumor stage is widely used for prognostication (4, 5). However, the system ignores other clinical features, which makes it difficult to represent the overall condition of the tumor and is highly heterogeneous. In recent years, studies have reported that molecular genetic markers, such as microsatellite instability and *K-ras*/*BRAF* mutation, are also related to the prognosis of CRC (28–30). These molecular genetic markers usually require complex and expensive laboratory techniques. Inflammation also plays a critical role in all stages of tumor progression (31–33). Multiple researchers have indicated the prognostic value of inflammation-related factors in CRC patients with different stages (34–36). The inflammatory process frequently causes changes in numerous hematological parameters, such as peripheral blood cell counts and levels of C-reactive protein and albumin. In comparison, peripheral blood cell counts are easy to measure, inexpensive, and widely available in routine clinical practice.

Several studies have examined the effects of markers, such as NLR, LMR, and PLR, with the results showing that high NLR, low LMR, and high PLR exhibited the worst OS in CRC (10–13, 37–39). However, these single markers were only based on the ratios of two types of blood cell counts, which might be influenced by various systemic factors and not accurately provide information on the process of inflammation. Therefore, a comprehensive blood biomarker is urgently needed in clinical practice.

Eosinophils and basophils, as rare sets of peripheral blood leukocytes, play important roles in tumors. Eosinophils are becoming recognized as a powerful immune effector and immunomodulator in the tumor microenvironment and have a potential role in tumor treatment (40). Basophils play a key role in various IgE-mediated and IgE-independent allergic inflammation (41). Studies showed that basophils released several angiogenic factors that play a pivotal role in inflammatory and tumor angiogenesis; histamine is released by basophils and has been implicated in CRC (42, 43). The cancer-changed immune cells in the tumor microenvironment have been reported to be closely related to the markers in peripheral blood (44). Eosinophils and basophils can be found not only in the tumor microenvironment but also in the blood. Previous studies have shown that tumor eosinophilia and basophilia infiltrations contribute to improving the survival of cancer patients (17, 45). Our study found that higher levels of circulating eosinophils and basophils in CRC tumors might be associated with better prognosis and survival, which is consistent with previous results. Therefore, we speculated that the combination of them in the blood might have a great predictive significance.

Our study, for the first time, comprehensively analyzed the 11 common inflammatory biomarkers based on eosinophil- and basophil-related markers, such as WER, PER, LER, NER, MER, EBR, WBR, PBR, LBR, NBR, and MBR; all provide certain prognostic values. To avoid the influence of multicollinearity, we performed the LASSO-Cox regression analysis and identified the nine valuable inflammatory markers to construct the RS. We found that RS was a significant independent prognostic factor in the training set. The prognostic value was validated in the entire cohort.

Previous research had demonstrated that 5-year relative survival rates for CRC patients range from more than 90% in stage I to slightly more than 10% in stage IV (4, 46). We could conclude that the prognosis of patients with stage I CRC was excellent, while patients with stage IV CRC have extremely poor prognoses; moreover, there were fewer patients with stage I and stage IV CRC. Therefore, the current study with a large scale and long follow-up time focused on stage II and stage III CRC patients. However, only relying on the tumor stage could not exactly predict the outcome for individual patients. It is necessary to construct novel prognostic markers with good performance. These markers could help quantify the risk of stage II and III CRC patients accurately. We constructed the RS based on eosinophil- and basophil-related markers in a large sample, and patients were classified into high-risk and low-risk groups, which is important for individualized risk stratification and timely intervention to improve prognosis. After the stratification of patients by subgroup, RS was still an independent prognostic factor for OS. According to the results of time-dependent ROC analysis, RS is superior to NLR, LMR, and PLR in prediction, and the combination of RS and tumor staging can improve the staging effect.

Nomogram is a practical graphical tool that is relatively easy to use and can assess the prognosis of individual patients (47). We tried to establish a prognostic nomogram to make it more intuitive and convenient to evaluate the prognosis of OS during clinical practice, and it can help CRC patients with poor prognosis to obtain better suitable treatment in advance. We developed a nomogram by incorporating RS and significant clinicopathological characteristics. The C-index and AUC of the nomogram were both higher than those for the staging system, which indicates a better prediction effect in the training and testing sets. Furthermore, the calibration curves of the 3-year and the 5-year probability of survival also demonstrated that the nomogram had good discrimination and calibration. Compared with staging, the nomogram also had a higher net benefit, which implies better clinical applicability of the nomogram.

Our study has several limitations that should be acknowledged. First, all the patients were selected from a single Chinese hospital; it will be better to validate the predictive accuracy of the model with external multi-center validation. Additionally, we mainly focused on the correlation between markers and OS in CRC; however, the dynamic changes and specificity of inflammatory markers were ignored. Furthermore, our study was a retrospective cohort, which comes with a limitation that some data on clinicopathological characteristics are lacking, such as other immune cells, tumor-infiltration eosinophils and basophils, microsatellite instability, and mismatch repair gene detection.

We integrated the accessible inflammatory markers based on eosinophils and basophils and constructed a novel RS in patients with stage II and III CRC. RS was shown to remain an independent factor for predicting the prognosis of CRC patients. Additionally, the nomogram developed by RS and clinicopathological characteristics might be used for the personalized prediction of CRC patients.

## Data availability statement

The raw data supporting the conclusions of this article will be made available by the authors, without undue reservation.

## Ethics statement

Ethical review and approval was not required for the study on human participants in accordance with the local legislation and institutional requirements. Written informed consent for participation was not required for this study in accordance with the national legislation and the institutional requirements.

## Author contributions

LG, YSZ, and YLL conceived and designed the study. LG, CYu, JF, TT, LZ, YL, SM, YPL, YYZ, JX, CJ, DZ, TZ, QF, ST, LL, and CYa performed the data collection. LG, JF, HH, DL, and LZ performed the statistical analysis. LG performed original draft writing, and JF, YZ, and YLL helped to review the manuscript. All authors read and critically revised the manuscript for intellectual content and approved the final manuscript.
