# Supplementary material for: Prognostic scoring system based on eosinophil- and basophil-related markers for predicting the prognosis of patients with stage II and stage III colorectal cancer: a retrospective cohort study
Source: Front Oncol. 2023 Jul 14;13:1182944. doi: 10.3389/fonc.2023.1182944 (PMC10375403; doi:10.3389/fonc.2023.1182944)
Supplement: Supplementary file 1 [file DataSheet_1.docx]

Supplementary Material

**Prognostic scoring system based on eosinophil-, and basophil-related markers for predicting the prognosis of patients with stage II and stage III colorectal cancer: a retrospective cohort study**

**Lijing Gao^1†^, Chao Yuan^2†^, Jinming Fu^1^, Tian Tian^1^, Hao Huang^1^, Lei Zhang^1^, Dapeng Li^1^, Yupeng Liu^1^, Shuhan Meng^1^, Ying Liu^1^, Yuanyuan Zhang^1^, Jing Xu^1^, Chenyang Jia^1^, Ding Zhang^1^, Ting Zheng^1^, Qingzhen Fu^1^, Shiheng Tan^1^, Li Lan^3^, Chao Yang^3^, Yashuang Zhao^1*^, Yanlong Liu^2*^**

^1^Department of Epidemiology, School of Public Health, NHC Key Laboratory of Etiology and Epidemiology (23618504), Harbin Medical University, Harbin, Heilongjiang Province, P.R. China

^2^Department of Colorectal Surgery, Harbin Medical University Cancer Hospital, Harbin, Heilongjiang Province, P.R. China

^3^Division of Chronic and Non-communicable Diseases, Harbin Center for Diseases Control and Prevention, Harbin, Heilongjiang Province, P.R. China

Lijing Gao^1†^ and Chao Yuan^2†^ have contributed equally to this work and share first authorship.

*** Correspondence:**Yashuang Zhao
[zhao_yashuang@263.net](mailto:zhao_yashuang@263.net)

Yanlong Liu
[liuyanlong1979@163.com](mailto:liuyanlong1979@163.com)


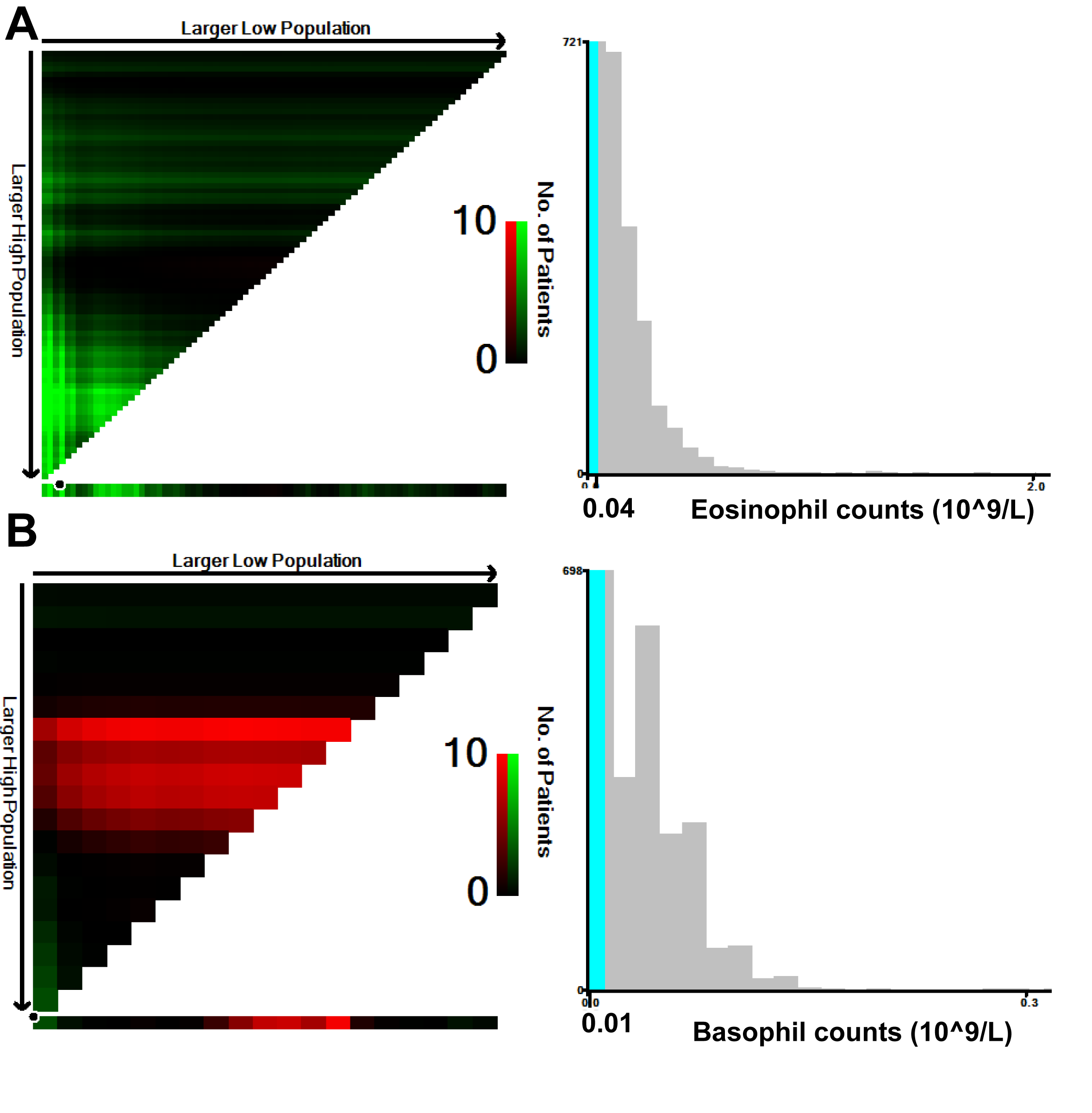


**Supplementary Figure 1. X-tile analysis of overall survival was performed to determine the cutoffs for eosinophil counts (A), and basophil counts (B)**

The optimal cutoffs for eosinophil counts and basophil counts were 0.04 (10^9/L) and 0.01 (10^9/L), respectively. X-tile plots are created by dividing marker data into two populations: low and high (left panels), red coloration of cut points indicates an inverse correlation with survival, whereas green coloration represents direct associations. The cut-point in the left panel is shown on a histogram of the training set (right panels).


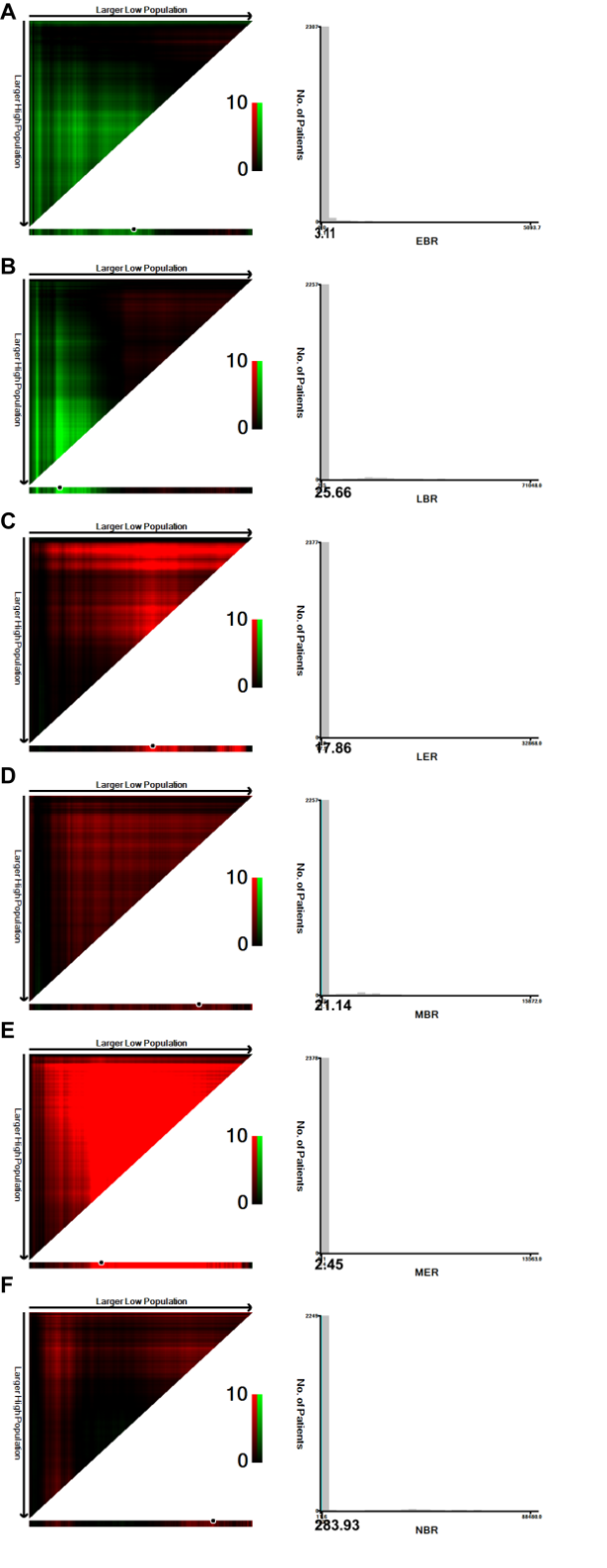


**Supplementary Figure 2. X-tile analysis of overall survival was performed to determine the cutoffs for EBR (A), LBR (B), LER (C), MBR (D), MER (E), and NBR (F)**

The optimal cutoffs for EBR, LBR, LER, MBR, MER, and NBR were 3.11, 25.66, 17.86, 21.14, 2.45, 19.50, and 283.93, respectively. EBR, eosinophil-to-basophil ratio; LBR, lymphocyte-to-basophil ratio; LER, lymphocyte-to-eosinophil ratio;MBR, monocyte-to-basophil ratio; MER, monocyte-to-eosinophil ratio; NBR, neutrophil-to-basophil ratio; X-tile plots are created by dividing marker data into two populations: low and high (left panels), red coloration of cut points indicates an inverse correlation with survival, whereas green coloration represents direct associations. The cut-point in the left panel is shown on a histogram of the training set (right panels).


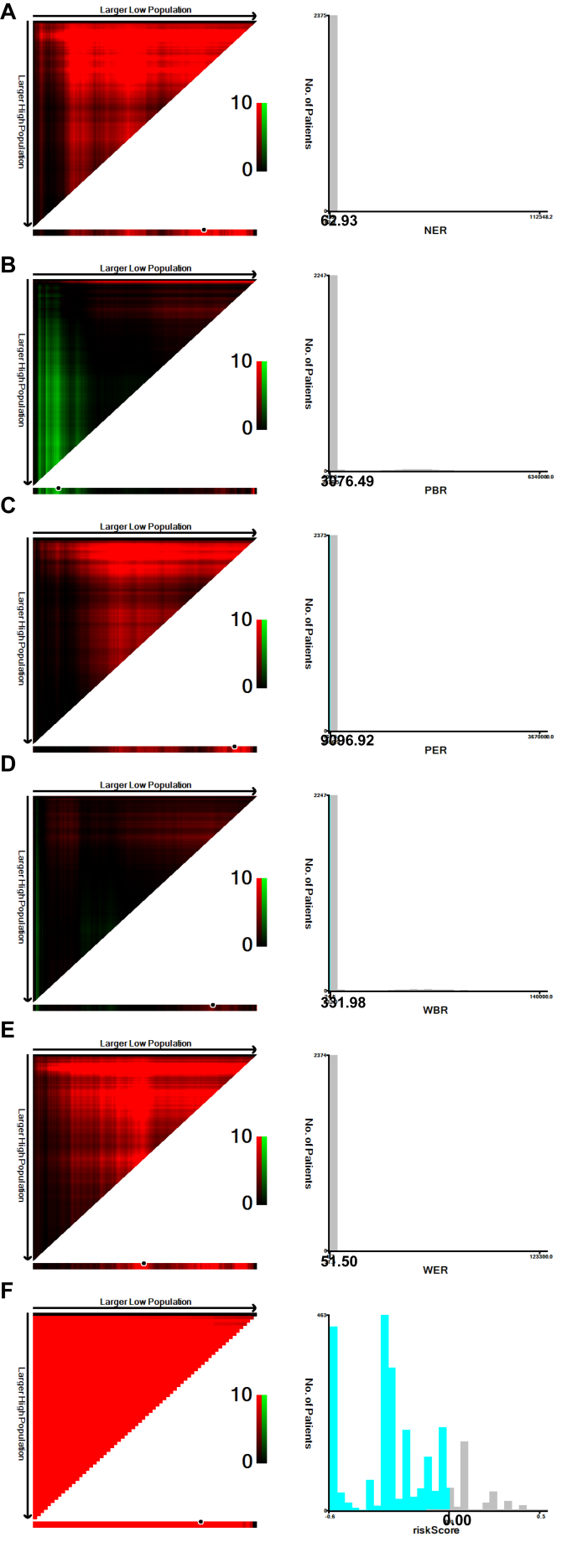


**Supplementary Figure 3. X-tile analysis of overall survival was performed to determine the cutoffs for NER (A), PBR (B), PER (C), WBR (D), and WER (E)**

The optimal cutoffs for NER, PBR, PER, WBR, and WER of OS were 62.93, 3076.49, 9096.92, 331.98, and 51.50, respectively. OS, overall survival; NER, neutrophil-to-eosinophi ratio; PBR, platelet-to-basophil ratio; PER, platelet-to-eosinophil ratio; WBR, white blood cell-to-basophil ratio; WER, white blood cell-to-eosinophil ratio; X-tile plots are created by dividing marker data into two populations: low and high (left panels), red coloration of cut points indicates an inverse correlation with survival, whereas green coloration represents direct associations. The cut-point in the left panel is shown on a histogram of the training set (right panels).


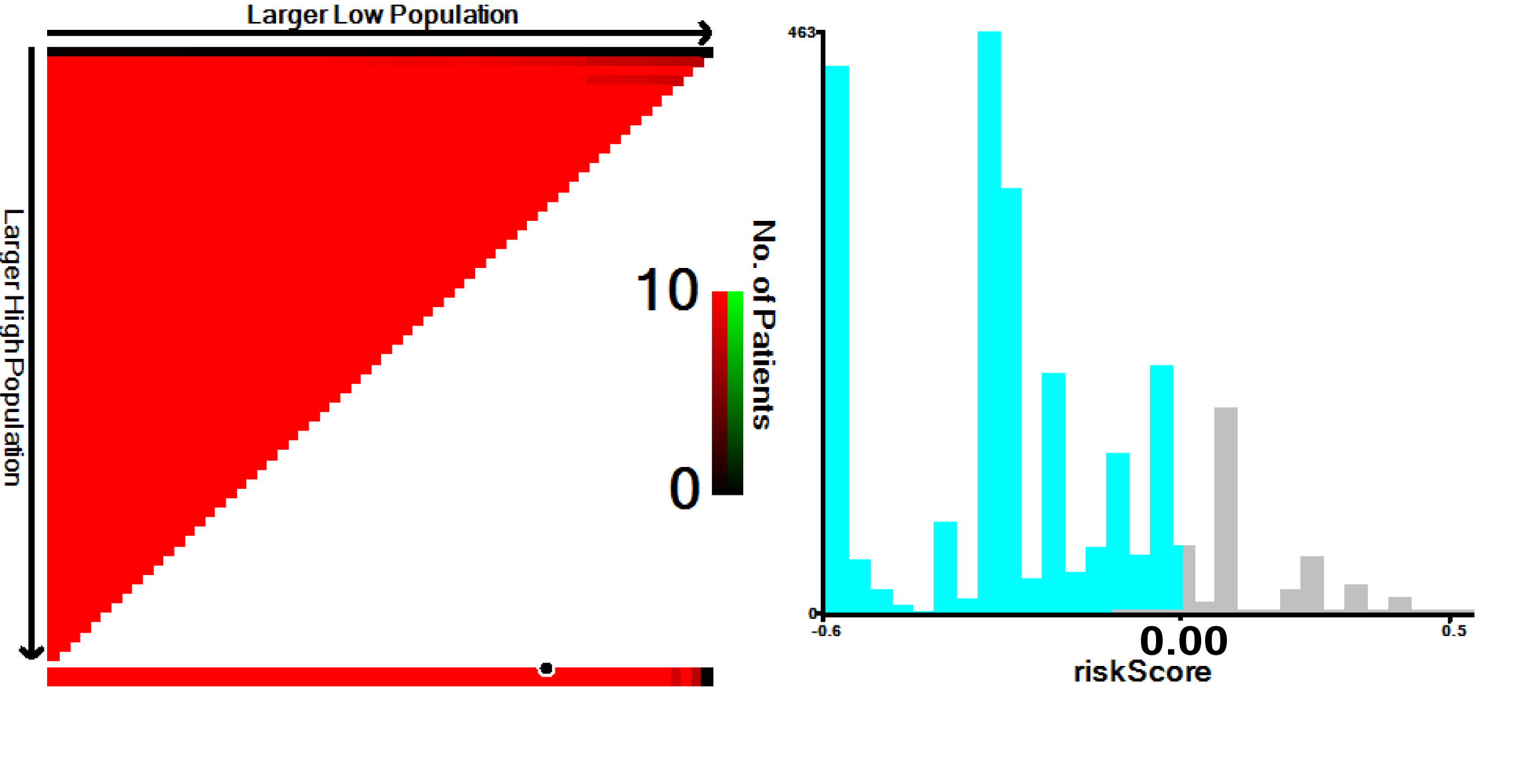


**Supplementary Figure 4. X-tile analysis of overall survival was performed to determine the cutoffs for risk score**

The optimal cutoffs for risk score is 0.00. X-tile plots are created by dividing marker data into two populations: low and high (left panels), red coloration of cut points indicates an inverse correlation with survival, whereas green coloration represents direct associations. The cut-point in the left panel is shown on a histogram of the training set (right panels).


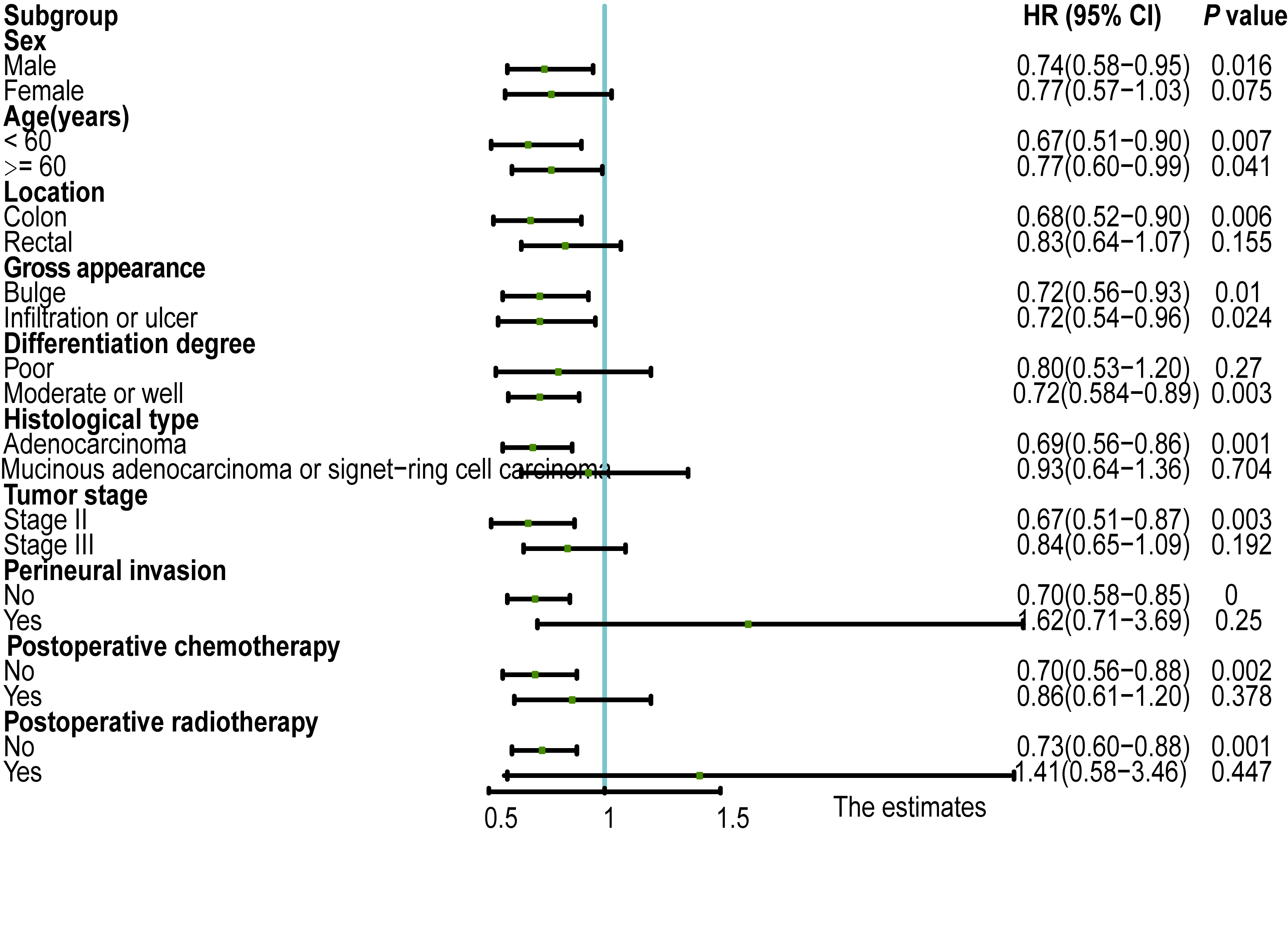


**Supplementary Figure 5. Association between eosinophils and overall survival by subgroup analysis in the training set**

**
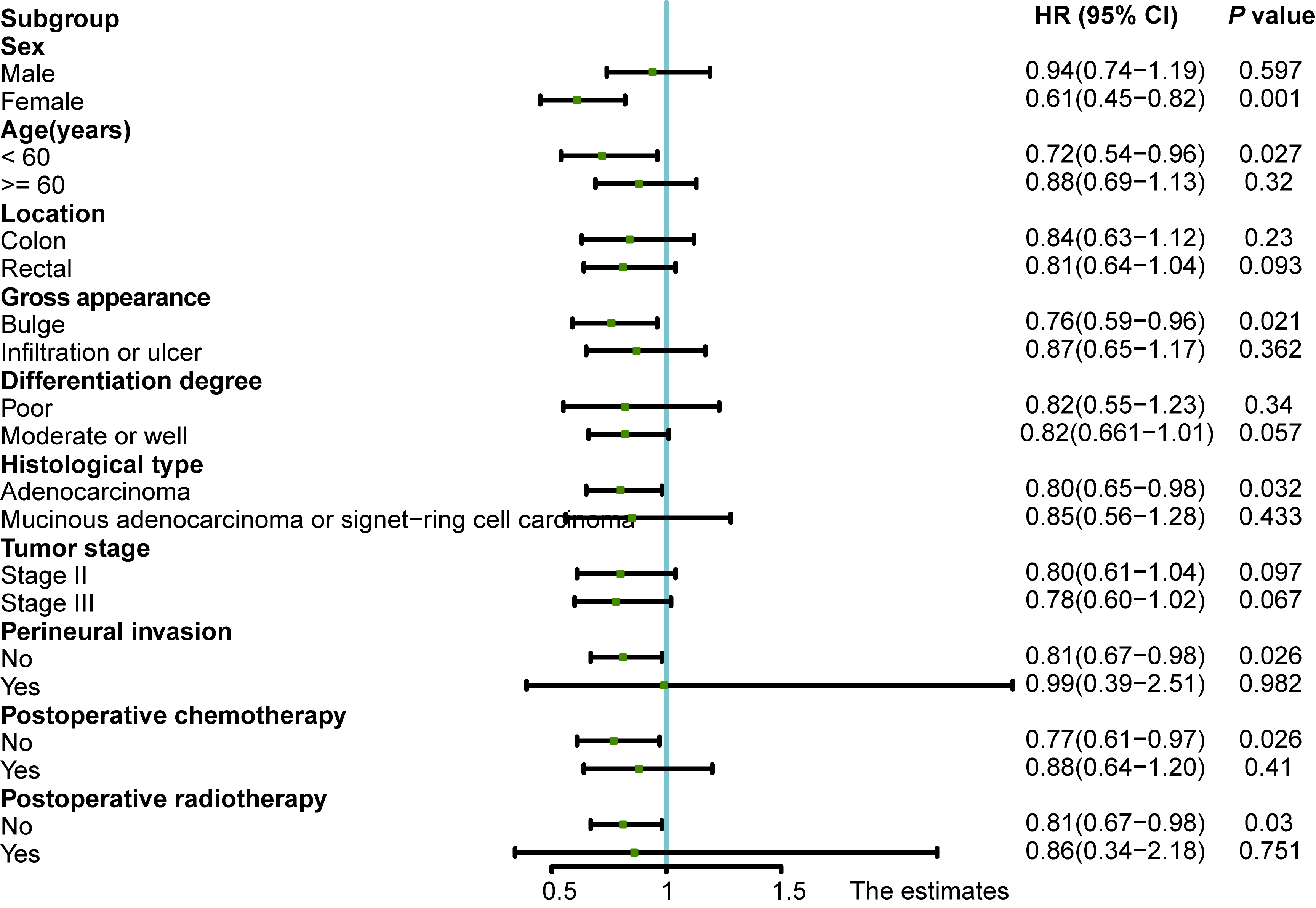
**

**Supplementary Figure 6. Association between basophils and overall survival by subgroup analysis in the training set**


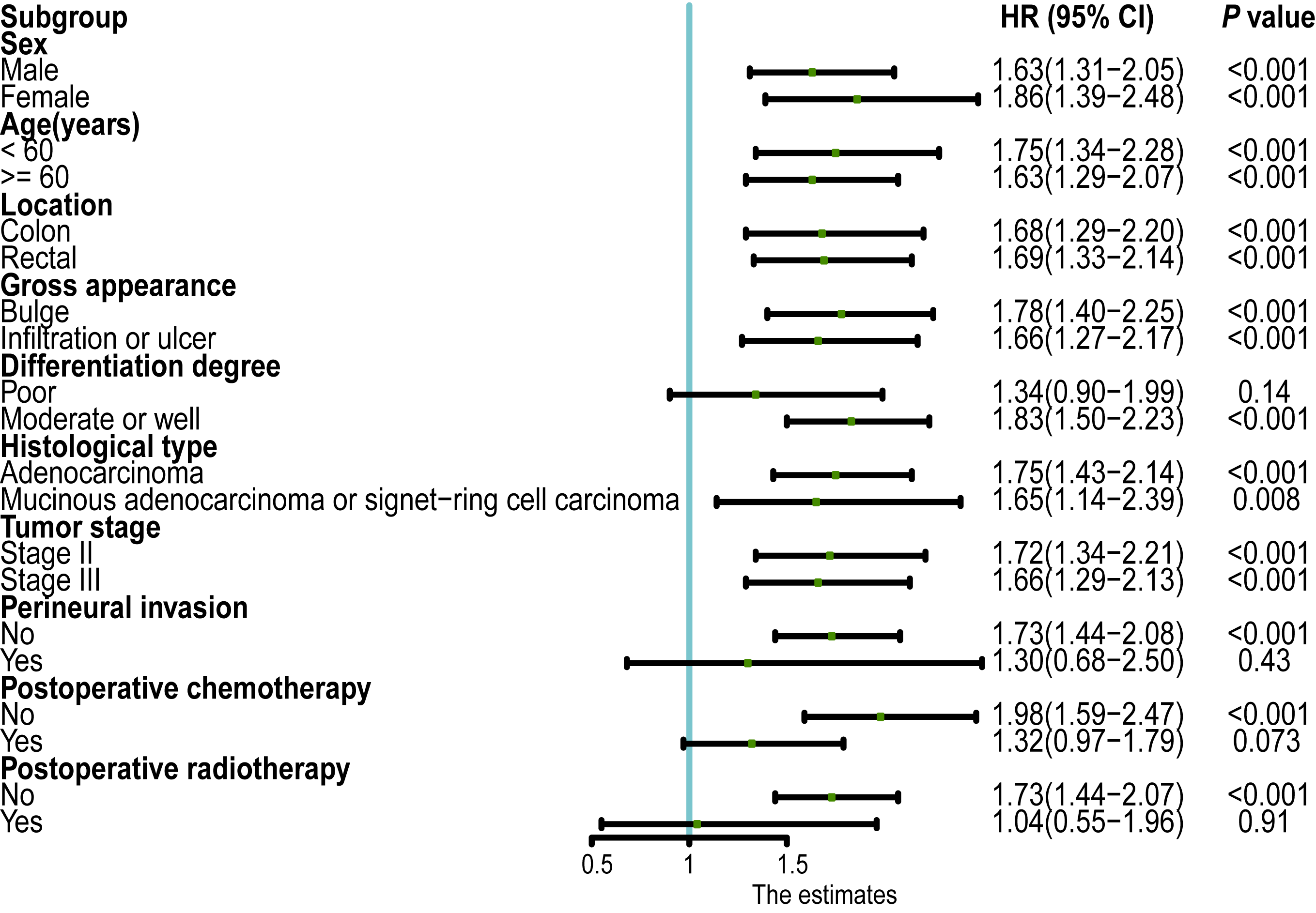


**Supplementary Figure 7. Association between risk score and overall survival by subgroup analysis in the training set**

**Supplementary Figure 8. Time-dependent ROC analyses of RS and other markers for predicting overall survival of patients with colorectal cancer in the training cohort**

**Abbreviations**: RS, risk score; NLR, neutrophil-to- lymphocytel ratio; LMR, lymphocyte-to-monocyte ratio; PLR, platelet-to-lymphocyte ratio;


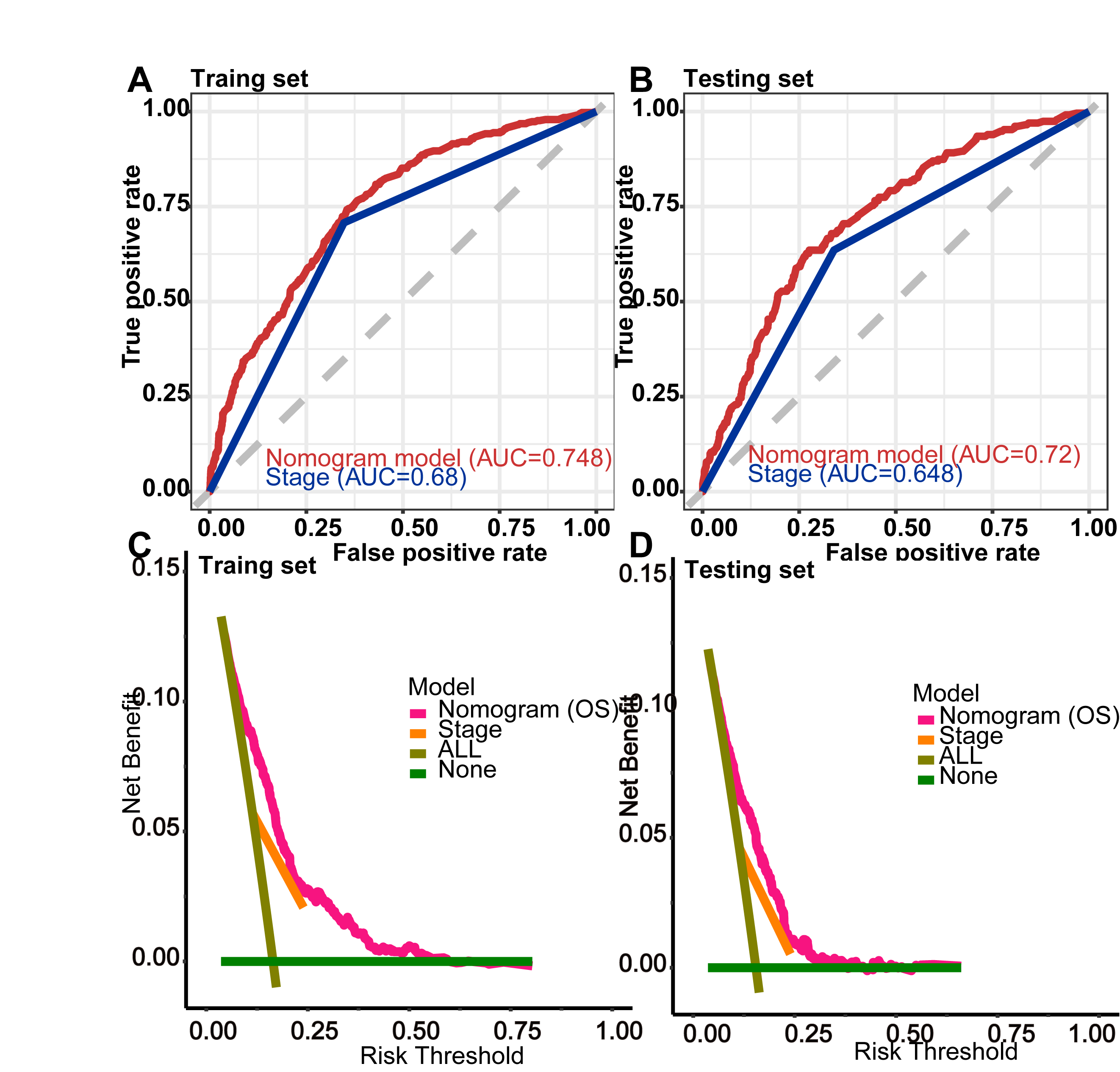


**Supplementary Figure 9. ROC analyses and decision curves of the nomogram and stage in the prediction of prognosis in the training and testing sets**

ROC analyses of the nomogram and stage in the prediction of prognosis at 3year in the training set (A) and testing set (B); Decision curve analysis for 3-year OS prediction in the training set (C) and the testing set (D).

**Supplementary Table 1 Univariate Cox analyses for OS of inflammation-related factors in the training set**

| **Variable** | **Cutoff value** | **Categories** | **HR** | **95% CI** | ***p*-value** |
| --- | --- | --- | --- | --- | --- |
| WER | 51.50 | High(>=51.50) vs.Low (<51.50) | 1.32 | 1.13-1.53 | <0.001 |
| PER | 9096.92 | High(>=9096.92) vs.Low (<9096.92) | 1.43 | 1.15-1.78 | 0.001 |
| LER | 17.86 | High(>=17.86) vs.Low (<17.86) | 1.29 | 1.11-1.50 | <0.001 |
| NER | 62.93 | High(>=62.93) vs.Low (<62.93) | 1.36 | 1.16-1.61 | <0.001 |
| MER | 2.45 | High(>=2.45) vs.Low (<2.45) | 1.44 | 1.21-1.70 | <0.001 |
| EBR | 3.11 | High(>=3.11) vs.Low (<3.11) | 0.82 | 0.71-0.95 | 0.010 |
| WBR | 331.98 | High(>=331.98) vs.Low (<331.98) | 1.21 | 1.02-1.44 | 0.026 |
| PBR | 3076.49 | High(>=3076.49) vs.Low (<3076.49) | 0.72 | 0.58-0.90 | 0.003 |
| LBR | 25.66 | High(>=25.66) vs.Low (<25.66) | 0.71 | 0.58-0.87 | <0.001 |
| NBR | 283.93 | High(>=283.93) vs.Low (<283.93) | 1.26 | 1.05-1.51 | 0.013 |
| MBR | 21.14 | High(>=21.14) vs.Low (<21.14) | 1.21 | 1.02-1.43 | 0.027 |

**Abbreviations**: HR = Hazard Ratio, CI = Confidence Interval; OS, overall survival; WER, (white blood cell)-to-eosinophil ratio; PER, platelet-to-eosinophil ratio; LER, lymphocyte-to-eosinophil ratio; NER, neutrophil-to-eosinophil ratio; MER, monocyte-to-eosinophil ratio; EBR, eosinophil-to--basophil ratio; WBR, (white blood cell)-to-basophil ratio; PBR, , platelet-to-basophil ratio; LBR, lymphocytel-to-basophil ratio; NBR, neutrophil-to-basophil ratio; MBR, monocyte-to-basophil ratio;

**Supplementary Table 2 The prognostic effects of eosinophils and basophils for patients with colorectal cancer in the training set**

| Marker | Univariate analyses | | |  | Multivariate analyses | | |
| --- | --- | --- | --- | --- | --- | --- | --- |
|  | HR | 95% CI | *p*-value |  | HR | 95% CI | *p*-value |
| Eosinophils (10^9/L) |  |  |  |  |  |  |  |
| < 0.04 | 1.00 |  |  |  | 1.00 |  |  |
| >=0.04 | 0.74 | 0.61-0.89 | 0.001 |  | 0.75 | 0.62-0.91 | 0.003 |
| Basophils (10^9/L) |  |  |  |  |  |  |  |
| <0.01 | 1.00 |  |  |  | 1.00 |  |  |
| >=0.01 | 0.85 | 0.71-1.02 | 0.087 |  | 0.81 | 0.67-0.98 | 0.027 |

**Abbreviation**: HR = Hazard Ratio, CI = Confidence Interval; All analyses are adjusted for sex, age, tumor location, gross appearance, differentiation degree, histologic type, tumor stage, cancer nodes, perineural invasion, neoplastic thrombosis, postoperative chemotherapy, and postoperative radiotherapy.

**Supplementary Table 3 Univariate and multivariate Cox analyses of baseline characteristics and risk score on survival in the entire set and testing set**

| Characteristic | All set | Univariate analyses | | | Multivariate analyses | | |  | Testing set | | Univariate analyses | | | Multivariate analyses | | |
| --- | --- | --- | --- | --- | --- | --- | --- | --- | --- | --- | --- | --- | --- | --- | --- | --- |
|  | N | HR | 95% CI | *p*-value | HR | 95% CI | *p*-value |  | N | HR | | 95% CI | *p*-value | HR | 95% CI | *p*-value |
| Sex | 3,986 |  |  |  |  |  |  |  | 1,595 |  | |  |  |  |  |  |
| Male |  | 1.00 |  |  | 1.00 |  |  |  |  | 1.00 | |  |  | 1.00 |  |  |
| Female |  | 0.83 | 0.74-0.94 | 0.003 | 0.80 | 0.70-0.90 | <0.001 |  |  | 0.78 | | 0.64-0.95 | 0.014 | 0.75 | 0.62-0.92 | 0.005 |
| Age | 3,986 |  |  |  |  |  |  |  | 1,595 |  | |  |  |  |  |  |
| < 60 |  | 1.00 |  |  | 1.00 |  |  |  |  | 1.00 | |  |  | 1.00 |  |  |
| >= 60 |  | 1.65 | 1.47-1.86 | <0.001 | 1.59 | 1.41-1.80 | <0.001 |  |  | 1.66 | | 1.37-2.00 | <0.001 | 1.51 | 1.24-1.84 | <0.001 |
| Location | 3,986 |  |  |  |  |  |  |  | 1,595 |  | |  |  |  |  |  |
| Colon cancer |  | 1.00 |  |  | 1.00 |  |  |  |  | 1.00 | |  |  | 1.00 |  |  |
| Rectal cancer |  | 1.3 | 1.15-1.46 | <0.001 | 1.19 | 1.05-1.35 | 0.005 |  |  | 1.28 | | 1.06-1.55 | 0.012 | 1.14 | 0.93-1.39 | 0.2 |
| Gross appearance | 3,986 |  |  |  |  |  |  |  | 1,595 |  | |  |  |  |  |  |
| Bulge |  | 1.00 |  |  | 1.00 |  |  |  |  | 1.00 | |  |  | 1.00 |  |  |
| Infiltration or ulcer |  | 1.53 | 1.36-1.72 | <0.001 | 1.41 | 1.25-1.59 | <0.001 |  |  | 1.63 | | 1.34-1.97 | <0.001 | 1.53 | 1.26-1.86 | <0.001 |
| Differentiation degree | 3,986 |  |  |  |  |  |  |  | 1,595 |  | |  |  |  |  |  |
| Poor |  | 1.00 |  |  | 1.00 |  |  |  |  | 1.00 | |  |  | 1.00 |  |  |
| Moderate or well |  | 0.65 | 0.56-0.75 | <0.001 | 0.72 | 0.62-0.84 | <0.001 |  |  | 0.74 | | 0.57-0.96 | 0.022 | 0.76 | 0.58-0.99 | 0.039 |
| Histological type | 3,986 |  |  |  |  |  |  |  | 1,595 |  | |  |  |  |  |  |
| Adenocarcinoma |  | 1.00 |  |  | 1.00 |  |  |  |  | 1.00 | |  |  | 1.00 |  |  |
| Mucinous adenocarcinoma or signet ring cell cancer |  | 1.15 | 1.00-1.31 | 0.047 | 1.31 | 1.14-1.50 | <0.001 |  |  | 1.01 | | 0.81-1.27 | 0.92 | 1.25 | 0.99-1.57 | 0.059 |
| Tumor_stage | 3,986 |  |  |  |  |  |  |  | 1,595 |  | |  |  |  |  |  |
| Stage II |  | 1.00 |  |  | 1.00 |  |  |  |  | 1.00 | |  |  | 1.00 |  |  |
| Stage III |  | 2.29 | 2.03-2.58 | <0.001 | 2.13 | 1.87-2.43 | <0.001 |  |  | 2.32 | | 1.92-2.81 | <0.001 | 2.03 | 1.64-2.51 | <0.001 |
| Cancer nodes | 3,986 |  |  |  |  |  |  |  | 1,595 |  | |  |  |  |  |  |
| No |  | 1.00 |  |  | 1.00 |  |  |  |  | 1.00 | |  |  | 1.00 |  |  |
| Yes |  | 2.43 | 2.00-2.95 | <0.001 | 1.45 | 1.18-1.79 | <0.001 |  |  | 3.04 | | 2.27-4.07 | <0.001 | 1.85 | 1.35-2.54 | <0.001 |
| Perineural invasion | 3,986 |  |  |  |  |  |  |  | 1,595 |  | |  |  |  |  |  |
| No |  | 1.00 |  |  | 1.00 |  |  |  |  | 1.00 | |  |  | 1.00 |  |  |
| Yes |  | 1.77 | 1.46-2.16 | <0.001 | 1.38 | 1.12-1.69 | 0.003 |  |  | 1.78 | | 1.28-2.49 | <0.001 | 1.36 | 0.96-1.93 | 0.081 |
| Neoplastic thrombosis | 3,986 |  |  |  |  |  |  |  | 1,595 |  | |  |  |  |  |  |
| No |  | 1.00 |  |  | 1.00 |  |  |  |  | 1.00 | |  |  | 1.00 |  |  |
| Yes |  | 2.32 | 1.78-3.03 | <0.001 | 1.55 | 1.18-2.04 | 0.002 |  |  | 2.69 | | 1.75-4.14 | <0.001 | 1.80 | 1.15-2.82 | 0.011 |
| Postoperative chemotherapy | 3,986 |  |  |  |  |  |  |  | 1,595 |  | |  |  |  |  |  |
| No |  | 1.00 |  |  | 1.00 |  |  |  |  | 1.00 | |  |  | 1.00 |  |  |
| Yes |  | 0.74 | 0.65-0.83 | <0.001 | 0.63 | 0.55-0.72 | <0.001 |  |  | 0.71 | | 0.58-0.86 | <0.001 | 0.59 | 0.48-0.72 | <0.001 |
| Postoperative radiotherapy | 3,986 |  |  |  |  |  |  |  | 1,595 |  | |  |  |  |  |  |
| No |  | 1.00 |  |  | 1.00 |  |  |  |  | 1.00 | |  |  | 1.00 |  |  |
| Yes |  | 2.08 | 1.67-2.60 | <0.001 | 2.03 | 1.61-2.55 | <0.001 |  |  | 2.31 | | 1.63-3.26 | <0.001 | 2.43 | 1.70-3.49 | <0.001 |
| Risk score | 3,986 |  |  |  |  |  |  |  | 1,595 |  | |  |  |  |  |  |
| Low |  | 1.00 |  |  | 1.00 |  |  |  |  | 1.00 | |  |  | 1.00 |  |  |
| High |  | 1.38 | 1.20-1.60 | <0.001 | 1.44 | 1.24-1.66 | <0.001 |  |  | 0.99 | | 0.77-1.27 | 0.920 | 1.07 | 0.83-1.38 | 0.600 |

**Abbreviation**: HR, hazard ratio; CI, confidence interval; All analyses are adjusted for sex, age, tumor location, gross appearance, differentiation degree, histologic type, tumor stage, cancer nodes, perineural invasion, neoplastic thrombosis, postoperative chemotherapy, and postoperative radiotherapy.

**Supplementary Table 4 Time-dependent ROC analyses of RS and other factors for predicting overall survival of patients with colorectal cancer in the training cohort.**

| AUROC | RS | TNM | TNM+RS | NLR | LMR | PLR |
| --- | --- | --- | --- | --- | --- | --- |
| 1-year | 0.628 | 0.604 | 0.661 | 0.659 | 0.412 | 0.656 |
| 2-year | 0.565 | 0.668 | 0.699 | 0.545 | 0.451 | 0.533 |
| 3-year | 0.587 | 0.679 | 0.720 | 0.542 | 0.464 | 0.523 |
| 4-year | 0.573 | 0.657 | 0.692 | 0.525 | 0.468 | 0.508 |
| 5-year | 0.571 | 0.650 | 0.685 | 0.527 | 0.470 | 0.497 |
| 6-year | 0.564 | 0.663 | 0.695 | 0.527 | 0.463 | 0.474 |
| 7-year | 0.564 | 0.685 | 0.722 | 0.535 | 0.459 | 0.469 |
| 8-year | 0.550 | 0.698 | 0.729 | 0.528 | 0.468 | 0.471 |
| 9-year | 0.554 | 0.708 | 0.745 | 0.509 | 0.476 | 0.463 |
| 10-year | 0.491 | 0.697 | 0.704 | 0.477 | 0.474 | 0.447 |
| 11-year | 0.475 | 0.710 | 0.710 | 0.444 | 0.462 | 0.382 |

**Abbreviations**: RS, risk score; NLR, neutrophil-to- lymphocytel ratio; LMR, lymphocyte-to-monocyte ratio; PLR, platelet-to-lymphocyte ratio;
